# Protective Effects of Gnetin C from Melinjo Seed Extract against High-Fat Diet-Induced Hepatic Steatosis and Liver Fibrosis in NAFLD Mice Model

**DOI:** 10.3390/nu15183888

**Published:** 2023-09-06

**Authors:** Tohfa Kabir, Haruki Yoshiba, Afifah Zahra Agista, Halima Sultana, Yusuke Ohsaki, Chiu-Li Yeh, Ryota Hirakawa, Hiroko Tani, Tomoki Ikuta, Tomonori Nochi, Suh-Ching Yang, Hitoshi Shirakawa

**Affiliations:** 1 Laboratory of Nutrition, Graduate School of Agricultural Science, Tohoku University, Sendai 980-8572, Japan; 2 International Education and Research Center for Food Agricultural Immunology, Graduate School of Agricultural Science, Tohoku University, Sendai 980-8572, Japan; 3 School of Nutrition and Health Sciences, Taipei Medical University, Taipei 11031, Taiwan; 4 Laboratory of Functional Morphology, Graduate School of Agricultural Science, Tohoku University, Sendai 980-8572, Japan; 5 Institute for Bee Products and Health Science, Yamada Bee Company, Inc., Okayama 708-0393, Japan

**Keywords:** NAFLD, resveratrol, gnetin C, hepatic fibrosis, hepatic steatosis

## Abstract

Nonalcoholic fatty liver disease (NAFLD), the most common form of chronic liver disease, can progress to hepatic steatosis, inflammation, and advanced fibrosis, increasing the risk of cirrhosis. Resveratrol, a natural polyphenol with antioxidant and anti-inflammatory properties, is beneficial in treating multiple metabolic diseases. Gnetin C, a resveratrol derivative obtained from Melinjo seed extract (MSE), shares similar health-promoting properties. We investigated the role of gnetin C in preventing NAFLD in a mouse model and compared it with resveratrol. Male C57BL/6J mice were fed a control diet (10% calories from fat), a high-fat choline-deficient (HFCD) diet (46% calories from fat) and HFCD diet supplemented with gnetin C (150 mg/kg BW·day^−1^) or resveratrol (150 mg/kg BW·day^−1^) for 12 weeks. Gnetin C supplementation reduced body and liver weight, and improved blood glucose levels and insulin sensitivity. Both gnetin C- and resveratrol reduced hepatic steatosis, with gnetin C also decreasing liver lipid content. Gnetin C and resveratrol ameliorated HFCD diet-induced hepatic fibrosis. The mRNA expression results, and western blot analyses showed that gnetin C and, to some extent, resveratrol downregulated fibrosis markers in the TGF-β1 signaling pathway, indicating a possible safeguarding mechanism against NAFLD. These results suggest that gnetin C supplementation may protect against lipid deposition and hepatic fibrosis.

## 1. Introduction

Non-alcoholic fatty liver disease (NAFLD) affects approximately 25% of the global population and is the leading cause of cirrhosis and hepatocellular carcinoma. It is characterized by the presence of steatosis in >5% of hepatocytes, associated with metabolic factors, and without excessive alcohol consumption [1]. NAFLD encompasses a range of liver diseases, from non-alcoholic fatty liver (NAFL) with lipid accumulation in the liver (steatosis), to a more advanced stage of non-alcoholic steatohepatitis (NASH) with inflammation and fibrosis (presence of collagen fibers in the extracellular matrix (ECM)) [2]. Most importantly, NASH can progress to hepatic cirrhosis and hepatocellular carcinoma, as well as increase the risk of other lifestyle-related diseases, such as diabetes and heart disease, thereby increasing the mortality risk [2,3,4]. Due to the lack of approved therapies for NAFLD, there is an increasing demand for pharmacological therapies [1]. The available treatment options mainly focus on promoting healthy lifestyle behaviors, such as healthy diets and regular physical activity [5,6].

Polyphenols, the most abundant antioxidants in the human diet, have gained interest as potential nutraceutical supplements for NAFLD due to their anti-inflammatory and antioxidative properties [7]. Resveratrol (RSV) (*trans*-3,5,40-trihydroxystilbene) is a polyphenolic compound found in over 70 plant species, particularly in grape skin and seeds [8]. Research into this compound has demonstrated a wide range of bioactivities, such as anti-microbial capacity, anti-oxidative, anti-inflammatory, hypotensive, hypolipidemic, and immunomodulatory effects [3,9,10,11]. RSV has exhibited efficacies in preventing and managing several diseases, like cancer, cardiovascular diseases, neurodegenerative diseases, diabetes, and obesity [8,9,10,11]. Therefore, several research groups have attempted to investigate the effects of RSV on the pathological complications related to NAFLD using different rodent models under varied experimental conditions. In this regard, it has been shown that RSV can mitigate hepatic steatosis (lipid deposition) [12], inflammation, and fibrosis [2] and improve insulin sensitivity [13]. The protective effects of RSV are thought to involve mechanisms such as enhancing the expression of *Cpt1* mRNA, the rate-limiting enzyme of fatty acid β-oxidation [14,15], and downregulating *Acc* [12,14], and *Fasn* [13], key enzymes in hepatic de novo lipogenesis. Additionally, RSV activates AMP-activated protein kinase (AMPK), a key metabolic regulator, contributing to the improvement of the NAFLD phenotype in both mice and rat models [13,14,16]. Moreover, RSV has been shown to inhibit hepatic stellate cell activation in CCl_4_-induced cirrhotic rats [17] and HFD-fed LPS-administered mice [2], resulting in the suppression of hepatic fibrosis in both cases.

**Figure 1:**
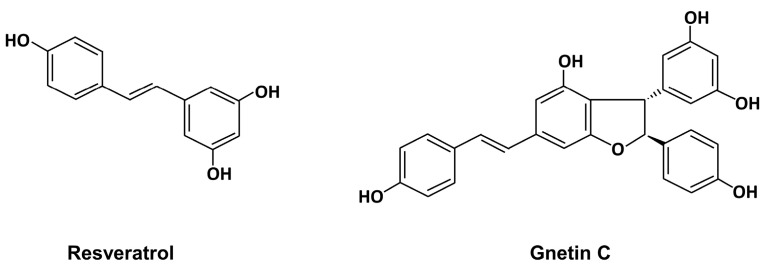
Chemical structure of resveratrol and gnetin C.

Melinjo (*Gnetum gnemon* L.) is a Gnetum family plant distributed from Southeast Asia to the Western Pacific region, and its seeds and fruits have been used as food since ancient times [18,19]. Apart from grapes, Melinjo seed extract (MSE) stands out as an abundant origin of RSV derivatives, including RSV, gnetin C, gnemonoside A (gnetin C diglucoside), and gnemonoside D (gnetin C monoglucoside). In the gastrointestinal tract, gnemonosides A and D are converted into gnetin C, rendering gnetin C as the major component of absorbed MSE [20,21]. The safety of MSE has been previously assessed in both rats and humans [22,23]. MSE has several pharmacological activities, including anti-microbial [20], antioxidant [24,25], anti-tumor [26,27,28,29], and immunomodulatory [24,30] effects. Furthermore, MSE has been shown to improve metabolic dysfunction in high-fat diet (HFD) models [31,32,33] and reduce liver weight in normal mice [34]. However, the effects of gnetin C and MSE in NAFLD remain unclear. The chemical structure of gnetin C resembles a dimer of RSV (Figure 1), and it is retained longer in the blood than RSV (mean residence time for gnetin C and RSV are 36.3 h and 6.6 h, respectively) [23], suggesting that purified gnetin C may have more advantageous effects against NAFLD.

Therefore, in this study, we evaluated the effects of MSE on the high-fat choline-deficient (HFCD) diet-induced NAFLD phenotype in mice. Following this, we investigated the preventive role of gnetin C against NAFLD in mice and compared its protective effects with RSV. Our study is the first to demonstrate the effects of gnetin C and RSV on HFCD-induced hepatic steatosis and fibrosis.

## 2. Materials and Methods

### 2.1. Preparation of MSE

The preparation of the Melinjo seed extract (MSE) for this experiment followed a previously documented procedure [21]. Briefly, Melinjo endosperms were acquired by drying and then removing the shells from the Melinjo seeds. The dried Melinjo endosperms were subsequently crushed and soaked in three times the volume of 50% ethanol at room temperature for a duration of 3 days. After this soaking period, the mixture underwent filtration. The resulting filtrate was then subjected to vacuum evaporation, resulting in the production of the MSE. The MSE powder used was standardized to contain no less than 20.0% RSV derivatives, which included 0.1% RSV, 2.5% gnetin C, 19.6% gnemonoside A, 4.3% gnemonoside D, and 9.0% dextrin.

### 2.2. Animals and Study Design

Male C57BL/6J mice were purchased from CLEA Japan Inc. (Tokyo, Japan), housed two animals per cage and maintained in a pathogen-free standard environment (room temperature: 23 ± 2 °C; humidity: 55 ± 10%; 12-h light/dark cycle). Ten-week-old mice were fed one of the three diets for 12 weeks: a control diet (10% calories from fat, A06071314M, Research Diet Inc., New Brunswick, NJ, USA), an HFCD diet (46% calories from fat, A06071318M, Research Diet Inc.), or HFCD + MSE diet (0.005%, 0.05%, or 0.5%). The details of the diet composition are shown in Table S1.

In another animal experiment, 10-week-old male C57BL/6J mice were divided into the following four groups: control, NAFLD, RSV, and gnetin C (n = 8 in each group); there was no significant difference in blood glucose level and body weight (BW) among the groups. The control group was fed the same control diet (A06071314M), the NAFLD group were fed the HFCD diet (A06071318M), and the RSV and gnetin C groups were fed the HFCD diet supplemented with either RSV (150 mg/kg BW·day^−1^) or gnetin C (150 mg/kg BW·day^−1^). Gnetin C and RSV were provided by the Yamada Bee Company, Inc. (Okayama, Japan). The administration dosages were determined based on 0.5% MSE supplementation relative to mice with an average initial body weight of 23 g and a daily diet intake of approximately 2.5 g (equivalent to 109 g/kg BW·day^−1^). Consequently, the average MSE consumption was 0.545 g/kg BW·day^−1^. The total gnetin C content in MSE was approximated to 26.4% and this led to consumption of gnetin C at 143.88 mg/kg BW·day^−1^ (26.4% of 0.545 g/kg BW·day^−1^, MSE). Thus, dosages of 150 mg/kg BW·day^−1^ were decided for gnetin C and RSV. BW and food intake were recorded weekly throughout the experimental period. After 12 weeks, the mice were euthanized after 5 h of fasting, and blood and tissue samples were collected. All animal experiments were conducted with the approval of the Animal Ethics Committee of Tohoku University. The approved document number is 2019AgA-011.

### 2.3. Blood Glucose, Insulin Tolerance Test (ITT), and Plasma Biochemical Parameters

Blood glucose levels were measured three times during the experimental period: at the start (0 week), 6th week, and 12th week using Nova StatStrip Express 900 (Siemens Healthcare, Co., Tokyo, Japan). An insulin-tolerance test was performed on the 10th week. After 3 h of fasting, insulin (Humulin R; Eli Lilly Japan K.K., Kobe, Japan) was injected intraperitoneally at a dose of 0.75 U per kg BW. Blood glucose levels were measured before (0 min) and 30, 60, 90, and 120 min after insulin administration. Plasma samples were prepared from the blood collected after euthanizing the mice on the 12th week. Plasma lipid profiles and liver function markers were analyzed by Oriental Yeast Co., Ltd. (Shiga, Japan). Plasma non-esterified free fatty acid (NEFA) levels were also measured using a laboratory assay NEFA kit (CAT# 633-52001; FUJIFILM Wako Pure Chemical Co., Osaka, Japan).

### 2.4. Measurement of Hepatic Lipid Content

Hepatic lipids were extracted according to the Folch’s method [35]. Briefly, approximately 100 mg of liver tissue was homogenized in 5 mL of chloroform/methanol (2:1) using a polytron homogenizer (PT2500E, Kinematica AG, Malters, Switzerland). The homogenate was filtered and transferred to another glass tube, followed by the addition of 1 mL of 0.05% H_2_SO_4_ and vertexing. After centrifugation for 10 min at 900× *g*, two phases appeared, and the lower organic phase was transferred to two other glass tubes (1 mL each), one for quantifying total/crude fat, and the other for estimating total cholesterol (TC), triglyceride (TG), and NEFA. Then, 1 mL of chloroform or 0.5% Triton X-100 was added to the organic phase, followed by evaporation. The residue was dissolved in distilled water, and hepatic TG, TC, and NEFA levels were measured using enzymatic methods (CAT#432-40201, #439-17501, and #633-52001; FUJIFILM Wako Pure Chemical Co.).

### 2.5. Liver Histopathology

For the histological analysis, liver tissue (0.1–0.2 g) was fixed in 10% formalin solution, dehydrated with various concentrations of ethanol solutions, and embedded in paraffin. Subsequently, the paraffin-embedded tissues were sectioned into 4 μm thicknesses for hematoxylin and eosin (H&E) as well as picrosirius red staining. The H&E- and picrosirius red-stained liver sections were examined using an Olympus IX81S1F-3 (Olympus, Tokyo, Japan), at 20× and 10× magnification of an objective lens, respectively. The images were analyzed using ImageJ software (version 1.37c, Wayne Rasband, National Institute of Health, Bethesda, MD, USA). The images from the H&E-stained liver sections were analyzed to quantify hepatic steatosis. Twelve to forty regions of interest (ROIs) were selected for each sample, and the fraction of empty circles (lipid droplets) was measured in all ROIs. Liver sections stained with picrosirius red were analyzed for hepatic fibrosis as indicated by the deposition of collagen fibers. Eight to sixteen ROIs from each sample were selected for analysis. The thresholds were adjusted equally for all images, and the fraction of the positive area (% of the collagen-stained area) was measured in all ROIs.

### 2.6. RNA Extraction and Quantitative Reverse Transcriptase-Mediated Polymerase Chain Reaction

Liver tissue preserved in RNA later was used for a quantitative reverse transcriptase-mediated polymerase chain reaction (RT-qPCR) following a previously described procedure [36]. The total RNA was extracted using the ISOGEN reagent (Nippon Gene, Co., Ltd., Tokyo, Japan) according to the manufacturer’s instructions. The quality and concentration of the extracted RNA were determined by measuring the absorbances at 260 and 280 nm wavelengths. The resulting RNA was denatured by incubating with 5 μM oligo-dT primers (Hokkaido System Science Co., Sapporo, Japan) and 1 mM dNTP (GE Healthcare, Tokyo, Japan) at 65 °C for 5 min. The denatured RNA was then used as a template to synthesize cDNA by mixing it with an RT buffer (50 mM Tris-HCl at pH 8.3, 75 mM KCl, 3 mM MgCl_2_, and 5 mM dithiothreitol) containing 50 U SuperScript III reverse transcriptase (Invitrogen, Carlsbad, CA, USA) and 20 U RNaseOUT RNase inhibitor (Invitrogen) and then incubating this mixture, followed by incubation at 50 °C for 60 min. The synthesized cDNA was used to amplify the target sequences using gene-specific primers (Table S2) and TB Green Premix Ex Taq (Takara Bio, Otsu, Japan) by qPCR in a CFX Connect real-time PCR detection system (Bio-Rad Laboratories, Inc., Hercules, CA, USA). The results were normalized to the eukaryotic elongation factor 1α1 (Eef1a1) level.

### 2.7. Enzyme-Linked Immunosorbent Assay

Plasma adiponectin levels were quantified using a commercially available mouse Adiponectin/Acrp30 ELISA kit (CAT#MRP 300; R&D Systems Inc., Minneapolis, MN, USA).

### 2.8. Western Blotting

Each mouse liver sample (approximately 100 mg) was homogenized in 1 mL of phosphate-buffered saline supplemented with a cOmplete protease inhibitor cocktail and PhosSTOP phosphatase inhibitor cocktail (Roche Applied Science, Mannheim, Germany), each tablet in 10 mL PBS according to the manufacturer recommendation using a polytron homogenizer at 10,000 rpm for 5 min with intervals. The intense homogenization, producing high-frequency pulses, results in the breakdown of subcellular organelles, such as mitochondria and the nucleus. As a result of this homogenization method, proteins can be extracted not just from the cytoplasm, but also from the nucleus [37]. The resulting tissue homogenate was centrifuged at 4 °C and 10,000× *g* for 20 min to obtain the protein lysate as a supernatant. The protein concentration in the lysate was measured at a wavelength of 595 nm by spectrophotometer using a Protein Assay Dye Reagent (Bio-Rad Laboratories, Inc.). Thereafter, an equal amount of protein (15 μg) for all the samples was resolved by electrophoresis in 12.5% sodium dodecyl sulfate polyacrylamide gel (FUJIFILM Wako Pure Chemical Co.). The separated proteins were transferred onto an Immobilon-P membrane (Millipore, Billerica, MA, USA). First, the membrane was incubated with a blocking buffer containing 3% bovine serum albumin (Sigma-Aldrich Japan K.K., Tokyo, Japan) prepared in TBS-T buffer (10 mM Tris-HCl at pH 7.5, 150 mM NaCl, and 0.1% Tween 20) for 1 h. Next, the membrane was incubated overnight at 4 °C with the following primary antibodies: phospho-AMPKα (CAT#2535; Cell Signaling Technology, Danvers, MA, USA) at a dilution of 1:10,000 and AMPKα (CAT#5831; Cell Signaling Technology) at 1:5000 dilution. The membrane was then incubated with the horseradish peroxidase-tagged secondary antibody at a dilution of 1:5000 for 1 h. The protein bands were visualized using an Immobilon western detection reagent (Millipore) and a ChemiDoc Imaging System (Bio-Rad Laboratories, Inc.). Similarly, an antibody against Pan-Actin (CAT#MS-1295, Thermo Fisher Scientific Japan, Tokyo, Japan) was also used at a dilution of 1:10,000. The images were analyzed using Image Lab 6.1 (Bio-Rad Laboratories, Inc.), and the relative protein expression levels were normalized to the amount of pan-actin.

### 2.9. Statistical Analysis

SigmaPlot v12.5 (Systat Software Inc., San Jose, CA, USA) was used for the statistical analysis, and the results were expressed as mean ± standard deviation (SD). At first, we checked all our data, whether normally distributed or not, using the Shapiro–Wilk test. If the data was normally distributed, we performed the Student’s *t*-test for a comparison between the control and NAFLD group, and one-way ANOVA followed by Dunnett’s post-hoc analysis among the three HFCD groups (NAFLD, gnetin C, and RSV). If some data failed the normality test, we conducted the Mann–Whitney rank sum test instead of the Student’s *t*-test and Kruskal–Wallis ANOVA on ranks instead of one-way ANOVA followed by Dunnett’s test. The statistical significance level was set at α = 0.05 (*p* < 0.05) and was indicated in each figure.

## 3. Results

### 3.1. MSE Ameliorated Hepatic Steatosis and Inflammation in NAFLD Model Mice

**Figure 2:**
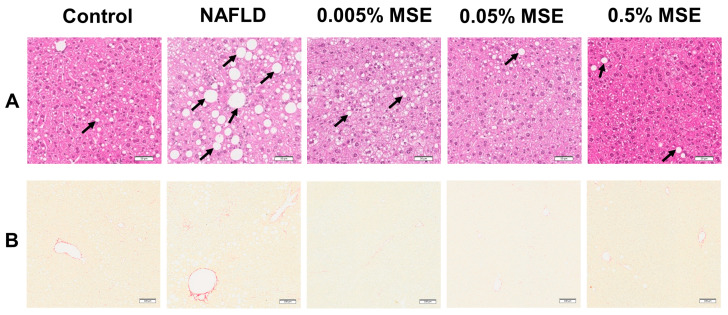
Representative histological images of (**A**) H&E-stained and (**B**) Picrosirius red-stained liver sections from mice fed with control diet (control), HFCD diet (NAFLD), HFCD + 0.005% MSE, HFCD + 0.05% MSE, and HFCD + 0.5% MSE. Images were taken using an Olympus IX81S1F-3 (Olympus, Tokyo, Japan), scale bar 50 μm and 100 μm for H&E-stained and Picrosirius red-stained images, respectively. Lipid droplets are indicated by black solid arrows (**A**) and liver fibrosis is identified by the presence of Picrosirius red-stained collagen fiber deposition (**B**).

Male C57BL/6J mice fed an HFCD diet for 12 weeks exhibited increased liver weight and elevated levels of liver injury markers (plasma activities of aspartate aminotransferase (AST), alanine aminotransferase (ALT), and alkaline phosphatase (ALP)). Steatosis and fibrosis were detected significantly by a histological analysis of the liver (Table 1) in comparison with the control diet. These results are consistent with previous findings [38], revealing that the HFCD diet induces NAFLD pathology. Mice supplemented with 0.5% MSE in the HFCD diet had significantly reduced body and liver weights compared to the NAFLD model mice, although there was no significant difference in food intake pattern between the NAFLD- and MSE-supplemented groups (Figure S1). Moreover, a dietary intake of 0.5% MSE significantly lowered plasma levels of TG, NEFA, and ALT, compared to the NAFLD mice. A liver histopathological analysis revealed that 0.05% and 0.5% MSE supplementation significantly attenuated steatosis (Figure 2A and Table 1). Additionally, dietary supplementation with 0.5% MSE tended to reduce HFCD diet-induced hepatic fibrosis in NAFLD mice (Figure 2B and Table 1).

### 3.2. Effect of Gnetin C and Resveratrol Supplementation on Body Weight, Feeding Efficiency, and Liver Weight in NAFLD Model Mice

**Figure 3:**
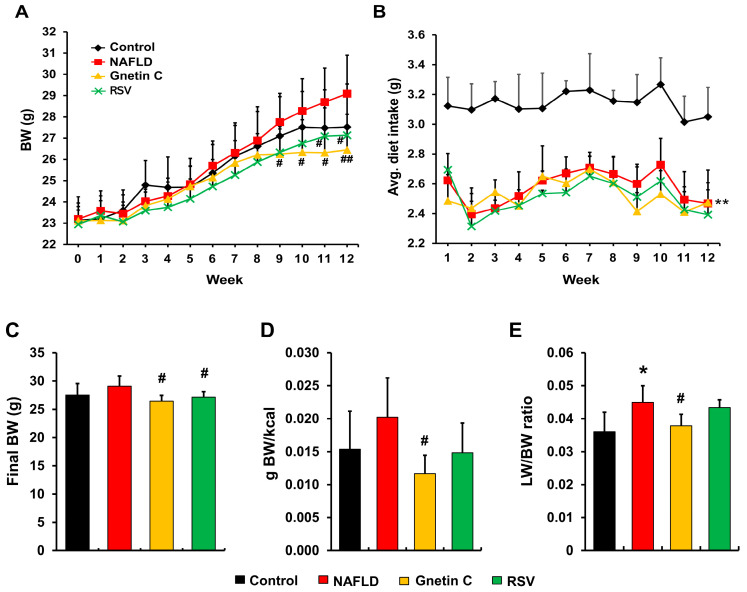
Gnetin C protects against high-fat diet-induced body weight and liver weight gain. (**A**) Body weight change curve for 12 weeks. (**B**) Weekly average diet intake. (**C**) Final body weight in week 12. (**D**) Feeding efficiency calculated from the total body weight gain and total calorie intake during the experimental period. (**E**) Liver weight adjusted by body weight. NAFLD, non-alcoholic fatty acid; BW, body weight; LW, liver weight. The data is presented as means ± SD, with n = 8. A Student’s *t*-test was used to compare the control and NAFLD groups for all the data presented in this section. Additionally, one-way ANOVA followed by Dunnett’s post-hoc analysis was carried out to make comparisons among the NAFLD, gnetin C, and RSV groups; * *p* < 0.05, ** *p* < 0.01 vs. control; ^#^ *p* < 0.05, ^##^ *p* < 0.01 vs. NAFLD.

To evaluate the therapeutic potential of gnetin C and resveratrol, the aforementioned NAFLD mice model was used. BW showed an increasing trend in the NAFLD group compared to the control group. However, during the last few weeks of the experimental period, gnetin C and RSV supplementation reduced BW compared to the NAFLD group (Figure 3A,C). Diet consumption was significantly higher in the control group than in the other three groups (Figure 3B), yet feeding efficiency was highest in the NAFLD mice and lowest in mice fed the gnetin C-supplemented diet (Figure 3D). At the end of week 12, the HFCD-fed NAFLD group showed a significant increase in the ratio of liver weight to BW compared with the control group, while gnetin C appeared to inhibit this increase in liver weight (Figure 3E).

### 3.3. Gnetin C Ameliorated Blood Glucose Rise and Improved Insulin Sensitivity

**Figure 4:**
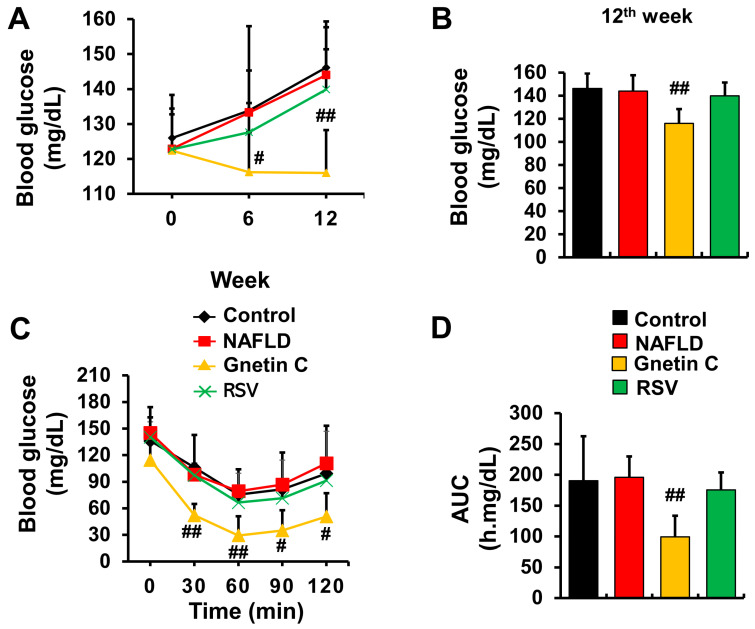
Gnetin C supplementation reduces blood glucose levels and improves insulin sensitivity. (**A**,**B**) Blood glucose levels measured thrice during the experimental period. (**C**,**D**) Insulin tolerance test (ITT) results and glucose response were calculated as the area under the curve (AUC). The data are presented as means ± SD, n = 7–8. To assess the differences between the control and NAFLD groups, we utilized the Student’s *t*-test for all the data in this figure. Additionally, we conducted one-way ANOVA followed by Dunnett’s post-hoc analysis to make comparisons among the NAFLD, gnetin C, and RSV groups; ^#^ *p* < 0.05, ^##^ *p* < 0.01 vs. NAFLD.

As NAFLD is associated with high blood glucose and insulin resistance, blood glucose levels were assessed at three time points (0, 6, and 12 weeks), and an ITT was performed at week 10. The blood glucose levels, and ITT analysis showed no difference between the control and NAFLD groups. However, dietary supplementation with gnetin C significantly reduced blood glucose levels at week 12 (Figure 4A,B) and improved insulin sensitivity (Figure 4C,D). These effects were not observed in the mice fed an RSV-supplemented HFCD diet.

### 3.4. Plasma Biochemical Parameters and Lipid Content

To assess the extent of liver damage caused by a HFCD diet, we analyzed liver damage markers in plasma, which are widely used in clinical diagnosis and are listed in Table 2. Plasma AST levels were significantly higher in all three HFCD-fed mice groups than in the control group. Compared to the control mice, an increase was observed in LDH (significantly) and ALT (tendency) levels in the NAFLD and RSV groups, but not in the gnetin C-supplemented group. Next, we estimated that the plasma lipid profile (Table 2) and the plasma levels of TG, TC, E-Cho, F-Cho, LDL-Cho, HDL-Cho, and NEFA were either significantly lower or tended to reduce in all three HFCD feeding groups compared to the control mice. Additionally, mice fed gnetin C-supplemented HFCD showed an even lower lipid content compared to the NAFLD and, to some extent, the RSV group. We also examined the plasma levels of adiponectin and found that its level was reduced by HFCD feeding in NAFLD mice (vs. the control group). However, dietary supplementation with gnetin C and RSV reversed this effect on plasma adiponectin levels (Table 2).

### 3.5. Effect of Gnetin C and Resveratrol Supplementation on HFCD-Induced Hepatic Steatosis and Inflammation

**Figure 5:**
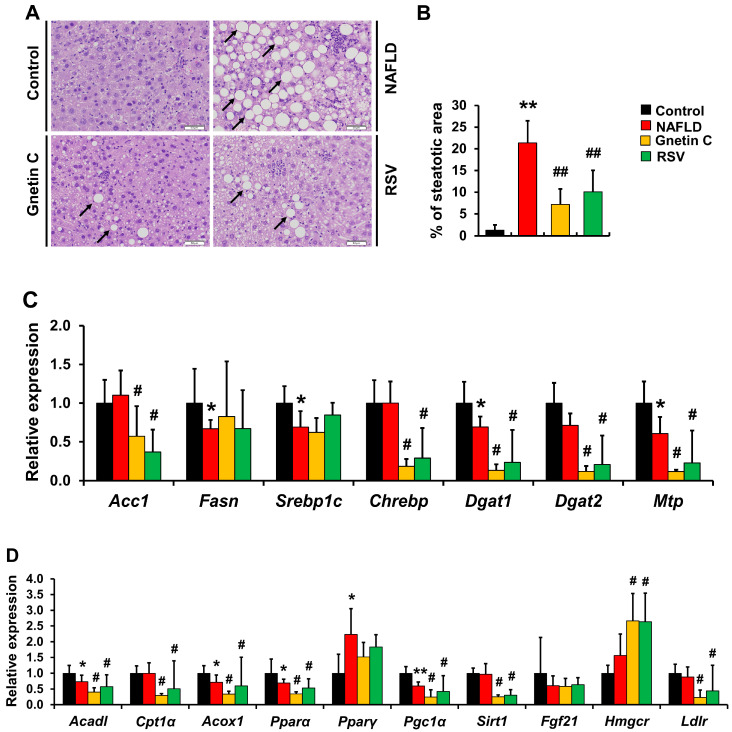
Dietary supplementation with gnetin C prevents hepatic steatosis induced by a high fat diet. (**A**) H&E-stained liver sections; images taken using Olympus IX81S1F-3 (Olympus, Tokyo, Japan) at 20× magnification of an objective lens and analyzed using ImageJ software. Lipid droplets are indicated by black. (**B**) Quantification of fat droplets as a measure of steatosis. (**C**) mRNA-expression levels of genes involved in fatty acid synthesis (*Acc1*, *Fasn*, *Srebp1c*, *Chrebp*) and triglyceride (TG) synthesis and transport (*Dgat1*, *Dgat2*, *Mtp*). (**D**) mRNA expression of genes involved in fatty acid β-oxidation (*Acadl*, *Cptα*, *Acox1*), lipid metabolism (*Pparα*, *Pparγ*, *Pgc1α*, *Sirt1*, *Fgf21*) and cholesterol synthesis (*Hmgcr*, *Ldlr*). Data are shown here as means ± SD; n = 7–8. A Student’s *t*-test was performed to compare control and NAFLD groups in case of steatosis and all mRNA data are presented here except for *Fgf21*, *Hmgcr*, and *Pparα.* mRNA levels of these three genes were analyzed using the Mann–Whitney rank sum test. For the comparison among NAFLD, gnetin C, and RSV groups, one-way ANOVA followed by Dunnett’s test was employed for the steatosis and mRNA levels of *Srebp1c*, *Pparγ*, *Sirt1*, *Fgf21*, and *Hmgcr*. Expression levels for all other genes (*Acc1*, *Fasn*, *Chrebp*, *Dgat1*, *Dgat2*, *Mtp*, *Acadl*, *Cptα*, *Acox1*, *Pparα*, *Pgc1α*, and *Ldlr*) were analyzed by Kruskal–Wallis ANOVA on ranks and Dunnett’s test for comparison among NAFLD, gnetin C, and RSV groups; * *p* < 0.05, ** *p* < 0.01 vs. control; ^#^ *p* < 0.05, ^##^ *p* < 0.01 vs. NAFLD.

H&E staining was performed to examine histopathological changes in the liver involved in steatosis. The results showed that a HFCD diet significantly increased the percentage of area covered by lipid droplets in NAFLD mice (vs. control mice), which was reversed by dietary supplementation with gnetin C and RSV (Figure 5A,B). Moreover, the mRNA-expression levels of hepatic inflammation-associated genes (*Il-6*, *Il-1β* and *Cd11b*) were evaluated and significantly decreased expression was observed only for the mRNA level of *Il-1β* in gnetin C and RSV-fed mice compared to the NAFLD mice (Figure S2). Furthermore, the liver lipid content (Table 2) showed elevated hepatic TG, TC, and total lipid levels in all three groups fed with a HFCD diet. These results suggested that mice in the NAFLD group experienced lipid accumulation in the liver. However, gnetin C intake tended to reduce the lipid content in the liver. Additionally, the NAFLD group exhibited significantly higher NEFA levels (vs. the control group), while gnetin C and RSV consumption suppressed this increase, further confirming the lipid-lowering effects of these two polyphenols.

### 3.6. Effects of Gnetin C and Resveratrol Supplementation on mRNA Expression of Genes Involved in Lipid Synthesis and Metabolism

Hepatic steatosis can develop from various mechanisms: increased NEFA uptake in the liver, lipid de novo synthesis, decreased fatty acid oxidation, increased TG storage, and decreased TG excretion from the liver via very-low-density lipoproteins (VLDL) [39]. Therefore, we evaluated which of these phenomena were upregulated in NAFLD model mice and subsequently downregulated by gnetin C and RSV interventions. We explored the expression of genes involved in lipid metabolism in the liver (Figure 5C,D). The mRNA expression of *Acc1* and *Chrebp*, two genes involved in fatty acid synthesis and regulation, respectively, were significantly reduced by gnetin C and RSV intervention (vs. NAFLD and control). In contrast, *Fasn* and *Srebp1c* mRNA levels were reduced in the NAFLD group (vs. the control group) but no change was observed in the gnetin C and RSV groups compared to the NAFLD group. Furthermore, significantly reduced mRNA expression in TG synthesis (*Dgat1*, *Dgat2*) and transport (*Mtp*) genes was observed in the gnetin C and RSV-supplemented groups compared to the NAFLD group. However, a decline in mRNA expression was noted among genes linked to fatty acid β-oxidation (*Acadl*, *Cpt1α*, *Acox1*) across all three HFCD-fed groups (NAFLD, gnetin C, and RSV). In addition, significant reductions were observed in lipid metabolism-related genes, *Pparα*, *Pgc1α*, and *Sirt1* (vs. the NAFLD group) in mice fed gnetin C- and RSV-supplemented diets. Furthermore, mRNA levels of *Pparα*, *Pgc1α*, and *Pparγ* were decreased or increased, respectively, in NAFLD mice liver (vs. control mice). The expression levels of *Fgf21* and *Pparγ* did not change after treatment with gnetin C or RSV. Also, the mRNA expression of *Hmgcr*, the rate-limiting enzyme in cholesterol synthesis, was significantly higher in the gnetin C and RSV groups than the NAFLD groups. Alternatively, the expression of *Ldlr* gene, a receptor for transporting blood LDL cholesterol into the liver, was lower in the gnetin C group (vs. NAFLD) but not in the RSV group.

### 3.7. Effect of Gnetin C and Resveratrol Supplementation in Suppressing Hepatic Fibrosis Progression

**Figure 6:**
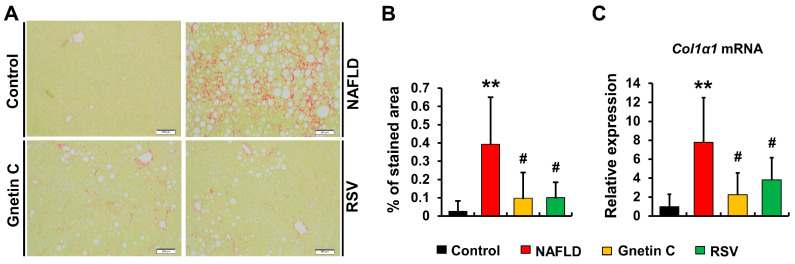
Dietary supplementation with gnetin C protects against high-fat diet-induced hepatic fibrosis. (**A**) Picrosirius red (PSR)-stained liver sections imaged at 10× magnification of an objective lens and analyzed using ImageJ software. (**B**) Quantification of collagen fiber deposition as an extent of hepatic fibrosis. (**C**) mRNA expression of Col1α1, the most common ECM protein. Data are shown here as means ± SD; n = 8; data obtained for collagen fiber deposition and mRNA levels of *Col1α1* were analyzed using the Mann–Whitney rank sum test to compare between control and NAFLD groups, while one-way ANOVA followed by Dunnett’s post hoc analysis was conducted for comparison among the NAFLD, gnetin C, and RSV groups; ** *p* < 0.01 vs. control; ^#^ *p* < 0.05 vs. NAFLD.

We examined the hepatic fibrosis phenotype by Picrosirius red staining of liver sections to determine collagen deposition levels. The results showed a significant increase in collagen fiber deposition in the NAFLD mice vs. the control mice, which was effectively attenuated by both gnetin C and RSV supplementation (Figure 6A,B). Subsequently, we analyzed the gene expression of the most abundant ECM protein *Col1α1*, as shown in Figure 6C. The HFCD diet markedly increased *Col1α1* mRNA expression in the NAFLD mice model (vs. the control group). However, compared with the NAFLD mice, this surge was significantly inhibited by gnetin C and RSV supplementation.

**Figure 7:**
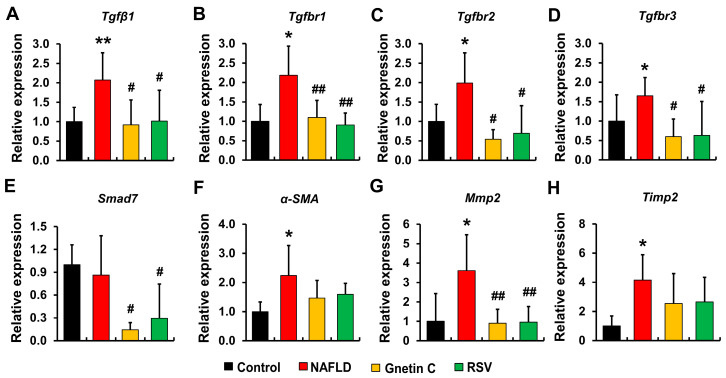
Effects of gnetin C supplementation on the TGFβ-signaling pathway. (**A**–**H**) mRNA expression of genes involved in the TGFβ-signaling pathway: (**A**) *Tgfβ1*, (**B**) *Tgfbr1*, (**C**) *Tgfbr2*, (**D**) *Tgfbr3*, (**E**) *Smad7*, (**F**) *α-SMA*, (**G**) *Mmp2*, (**H**) *Timp2*. Data are shown here as means ± SD; n = 8, a Student’s *t*-Test was performed to compare control and NAFLD groups for of *Tgfβ1*, *Tgfbr1*, *Tgfbr2*, *Smad7*, *α-SMA*, and *Timp2* mRNA levels and a Mann–Whitney rank sum test was conducted for *Tgfbr3* and *Mmp2* mRNA levels. For the comparison among NAFLD, gnetin C, and RSV groups, one-way ANOVA followed by Dunnett’s test was employed for the mRNA levels of *Tgfβ1*, *Tgfbr1*, *α-SMA*, *Mmp2*, and *Timp2* and Kruskal–Wallis ANOVA on ranks and Dunnett’s test were conducted for the mRNA levels of *Tgfbr2*, *Tgfbr3*, and *Smad7*, for comparison among the NAFLD, gnetin C, and RSV groups; * *p* < 0.05, ** *p* < 0.01 vs. control; ^#^ *p* < 0.05, ^##^ *p* < 0.01 vs. NAFLD.

Next, we analyzed the mRNA expression of several genes associated with the TGF-β1-mediated signaling pathway that leads to fibrotic tissue scaring in the liver. We found that the mRNA expression of *Tgfβ1* along its three receptors, *Tgfbr1*, *Tgfbr2*, and *Tgfbr3*, was enhanced by HFCD feeding in NAFLD mice compared to that of the control group (Figure 7A–D). On the contrary, the elevated expression of these fibrogenic genes was repressed by dietary supplementation with gnetin C and RSV. Additionally, mRNA levels of α-smooth muscle actin protein (*α-SMA*), matrix metalloproteinase 2 (*Mmp2*), and tissue inhibitors of matrix metalloproteinase 2 (*Timp2*), shown in Figure 7F–H were significantly higher in the NAFLD mice (vs. control mice), but not in gnetin C- and RSV-supplemented mice, reflecting an imbalance in ECM protein synthesis and degradation caused by the HFCD diet. In contrast, a further reduction in the expression of *Mmp2* was observed in the livers of mice fed the gnetin C- and RSV-supplemented diets. We also examined the mRNA expression of the antifibrogenic marker *Smad7*, which was significantly lower in both gnetin C and RSV groups than in the control and NAFLD groups (Figure 7E). These findings also suggest that gnetin C and RSV exert fibrosis-inhibitory effects.

### 3.8. Effect of Gnetin C and Resveratrol Supplementation on AMPK Activation

**Figure 8:**
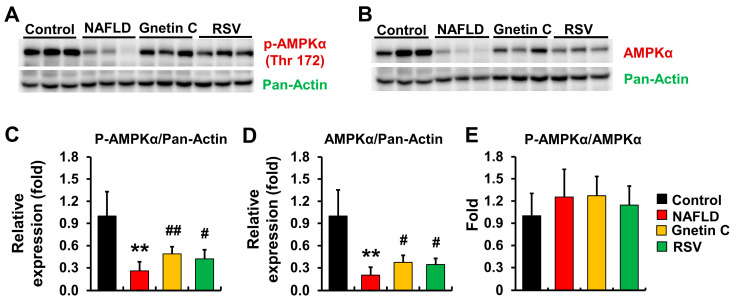
Effect of gnetin C supplementation on the hepatic expression of AMPKα and phosphorylated AMPKα (p-AMPKα) protein. (**A**,**B**) Representative Western blot images for the protein expression of (**A**) p-AMPKα and (**B**) AMPKα. (**C**–**E**) Quantification of the protein expression of (**C**) p-AMPKα, (**D**) AMPKα, and (**E**) ratio of p-AMPK to total AMPK. Data are shown here as means ± SD; n = 8. Values were normalized by the hepatic expression of Pan-Actin as an internal standard. The relative protein expression of p-AMPK and the ratio of p-AMPK to total AMPK were evaluated by the Student’s *t*-Test, while the total AMPK level was evaluated by the Mann–Whitney rank sum test to compare between control and NAFLD groups. One-way ANOVA followed by Dunnett’s analysis was conducted for comparison among the NAFLD, gnetin C, and RSV groups for p-AMPK, total AMPK, and the p-AMPK/AMPK ratio; ** *p* < 0.01 (vs. control), ^#^ *p* < 0.05, ^##^ *p* < 0.01 (vs. NAFLD).

We examined the protein levels of total and phosphorylated AMPK using Western blotting because AMPK is thought to be involved in the development of NAFLD through several mechanisms. One of the proposed mechanisms is the TGF-β1-mediated signaling pathway, where activated AMPK (p-AMPK) might inhibit the transcription coactivator P300, thereby blocking the transcriptional activation of the genes that trigger fibrogenesis [40]. In this study, reduced levels of both AMPK and p-AMPK were observed in HFCD-fed NAFLD model mice (Figure 8A–D). However, a significant increase in the expression of AMPK and p-AMPK was observed in the gnetin C- and RSV-supplemented groups vs. the NAFLD group. However, the ratio of p-AMPK to total AMPK was not different among the groups (Figure 8E).

## 4. Discussion

In this study, we showed for the first time that MSE administration repressed liver fattening and other liver histopathological features (steatosis and fibrosis) induced by a HFCD diet. Although information regarding the effects of MSE in ameliorating NAFLD is insufficient, some researchers have identified its anti-obesity effects, showing that dietary supplementation with MSE potentially reduces HFD-induced BW gain, hepatic TG content, and improves insulin resistance in mice [31,32]. Based on these results and our own, we speculated that the beneficial effects of MSE might arise from its constituents, RSV and gnetin C. It has also been observed in this experiment that 0.5% MSE supplementation prevented HFCD diet-induced body weight gain, showing the lowest body weight among all the groups studied. In this instance, we suggest that the 0.5% MSE-induced weight reduction is attributed to the calorie restriction mimetic effects of RSV and its derivatives present in MSE [31]. Hence, the inference drawn is that a 0.5% MSE supplementation could serve as the efficacious dose to improve the NAFLD phenotype induced by HFCD. Additionally, it becomes possible to calculate the human equivalent dosage (HED) for 0.5% MSE using an animal-to-human dose conversion equation [41]. For an individual weighing 60 kg, the appropriate MSE dosage would be 2652 mg/day, accounting for the variations in metabolic rates and body dimensions between mice and humans. Next, we focused on its major constituent, gnetin C, which is presumed to have beneficial effects against NAFLD development. To gain deeper insights into its efficacy, we compared its application as a dietary intervention with that of RSV. Our results demonstrated that supplementing gnetin C to the HFCD diet can effectively protect mice from developing NAFLD pathology by reducing liver weight, BW, and blood glucose levels, improving insulin sensitivity, and inhibiting liver steatosis and fibrosis. It is worth mentioning that, in this study, gnetin C exhibited greater efficiency in protecting against NAFLD than RSV.

In our study, we used a high-fat diet with a complete absence of choline, but not methionine, to induce NAFLD pathology because the complete absence of both choline and methionine might result in weight loss even with the HFD [42]. In this study, we utilized a choline-deficient high-fat diet supplemented with 0.2% methionine (approximately 0.5% methionine in the control diet) (Table S1) to induce NAFLD pathology without weight loss [38]. Methionine and choline are involved in phosphatidylcholine (PC) metabolism, an essential component of VLDL required for TG excretion from the liver [43,44]. Therefore, feeding a choline-methionine-restricted diet results in lipid accumulation in the liver, leading to a more progressive NAFLD pathology within a short period. Therefore, the liver’s ability to excrete lipids is hindered by this phenomenon. As a result, this diet contributes to lowering the overall levels of lipids in the bloodstream. This pattern has been witnessed in our animal experiments (Table 1 and Table 2) and was also noted in a previous research study [37]. The progression of NAFLD pathology is considered a suitable model for screening drugs or food ingredients for the treatment of fatty liver disease. Under our experimental conditions, mice fed the HFCD diet for 12 weeks did not develop insulin resistance (IR) or obesity, consistent with a previous finding [38]. It is possible that increased methionine content and a longer feeding period may be needed to induce IR and obesity.

Although the detailed pathophysiology of NAFLD remains poorly understood, it has been characterized by the presence of lipid droplets and inflammatory cell infiltration in the liver, which are visible under a light microscope. However, in our animal model, supplementation with gnetin C and RSV protected against developing such histological features. Based on the mRNA expression data (Figure 5C), we hypothesized that gnetin C and RSV may prevent lipid accumulation in the liver by inhibiting *Acc1* transcript levels, a key enzyme involved in de novo fatty acid synthesis by converting acetyl-CoA to malonyl-CoA. The primary lipids stored in the fatty liver are TGs synthesized by two diacylglycerol acyltransferases, Dgat1 and Dgat2. Gluchowski et al. indicated that the liver-specific knockdown of *Dgat2* reduced diet-induced hepatic steatosis [45]. Similarly, our dietary intervention with gnetin C and RSV resulted in a significant reduction in the mRNA levels of both *Dgat1* and *Dgat2*. These results suggest that the protective effects of gnetin C and RSV against hepatic steatosis may be mediated through the inhibition of lipogenesis and fat storage in the liver.

Previous studies have demonstrated that RSV can directly activate AMPK in neurons [46] and hepatocellular carcinoma HepG2 cells [47]. Another study reported the potential role of RSV in ameliorating NAFLD by activating AMPK in both cultured HepG2 cells and the liver of an NAFLD rat model [13]. Activated AMPK may improve NAFLD by reducing the expression of lipogenesis-associated genes, *Srebp1c*, *Fasn,* and *Acc1* [13,48]. Additionally, AMPK activation also contributes to the improvement of glucose homeostasis by enhancing glucose metabolism [13,49]. In our study, enhanced p-AMPK and total AMPK protein expression was detected in gnetin C- and RSV-supplemented mice compared to NAFLD mice (Figure 8). These enhanced AMPK levels may mediate the lipid-lowering effects of gnetin C and RSV. Furthermore, the improvements in blood glucose levels and insulin sensitivity induced by dietary gnetin C uptake, but not RSV, might also be mediated through AMPK activation.

Liver fibrosis represents a more progressive form of NAFLD that occurs if hepatic steatosis and inflammation persist for an extended period, leading to permanent damage to the liver [50]. If NAFLD progression remains untreated, permanent fibrotic damage can eventually promote hepatic cirrhosis and hepatocellular carcinoma. In our study, we also observed significant levels of fibrotic changes in the livers of mice fed the HFCD diet. However, this phenotype was ameliorated in the HFCD diet-fed mice supplemented with gnetin C and RSV. We also observed the significant transcriptional inhibition of *Col1α1*, a potent fibrogenic marker, in gnetin C supplemented mice liver.

Subsequently, we attempted to deduce the molecular mechanisms by which gnetin C and RSV may influence fibrosis progression in the liver. The prevention of liver fibrosis was found to be associated with the inhibition of the TGF-β1-signaling pathway. TGF-β1 is considered a critical inducer of liver fibrosis [51], and the severity of hepatic fibrosis is correlated with enhanced TGF-β1 expression [52,53]. Hepatic injury caused by lipid accumulation in the liver leads to the release of TGF-β1 from necrotic hepatocytes, which, in turn, activates adjacent hepatic stellate cells, resulting in the increased production of ECM proteins, of which collagen type I is the most dominant [51,54]. An intracellular signal is initiated upon the binding of TGF-β1 to its three cell surface receptors, TGFβRI, II, and III. Briefly, TGFβ binds to either TGFβRII directly, or TGFβRIII, which then presents TGFβ to the auto phosphorylated TGFβRII. Once activated by the ligand (TGFβ), TGFβRII forms a complex with TGFβRI, followed by the phosphorylation and activation of SMAD2/SMAD3 by the kinase activity of TGFβRI [55]. Next, activated SMAD2/SMAD3 forms an oligomeric complex with SMAD4, and this complex, along with the transcription coactivator P300, translocate into the nucleus to regulate the transcription of the target genes SMAD7, collagen, and α-SMA [40,51,54,56,57]. SMAD7 acts as a negative regulator of this signaling pathway, either by inhibiting the phosphorylation of SMAD2/SMAD3 or by promoting the ubiquitin-mediated degradation of SMAD2/SMAD3 and TGFβRI [54]. In our study, the RT-qPCR results showed increased hepatic expression of *Tgfβ1* along with its receptors *Tgfbr1*, *Tgfbr2*, and *Tgfbr3* in HFCD diet-fed NAFLD model mice. However, this elevated expression of the fibrogenic markers was successfully inhibited by gnetin C and RSV supplementation, suggesting a potential role of these two polyphenols in suppressing the master inducer of fibrogenesis.

Matrix metalloproteinases (MMPs) are involved in matrix protein degradation and their activities are regulated by specific inhibitors called TIMPs (tissue inhibitors of matrix metalloproteinase). In healthy livers, there is an equilibrium between the expression of MMPs and TIMPs to maintain ECM homeostasis [58,59]. However, during fibrogenesis in pathological conditions, the TGF-β1-mediated activation of hepatic stellate cells disrupts this equilibrium, leading to an increased amount of matrix proteins in extracellular space [54]. In our study, elevated mRNA levels of *Mmp2* and *Timp2* were observed in the livers of NAFLD model mice, reflecting the fibrogenic response induced by the HFCD diet; however, this elevation was reversed or trended to reverse in the livers of gnetin C- and RSV-supplemented mice. Additionally, there was a marked reduction in the expression of the anti-fibrogenic factor *Smad7* in gnetin C- and RSV-fed mice livers. These results indicate the preventive potential of gnetin C and RSV by inhibiting the upstream fibrogenic factors of the TGF-β1-signaling pathway, thus, repressing downstream markers as well.

Several studies have also suggested the role of adipokines in regulating hepatic fibrosis, with adiponectin being considered a potent anti-fibrogenic adipokine [60,61]. Adiponectin secreted from adipose tissue binds to its receptors on hepatic stellate cells during fibrogenesis, leading to the activation of AMPK. This activated AMPK may play a role in suppressing hepatic fibrosis by regulating transcriptional coactivator P300 activity, thus inhibiting the TGF-β1-signaling pathway [40,60]. A previous study suggested that RSV could increase circulating adiponectin levels in high-fat, high-carbohydrate diet-fed mice [62]. Another study revealed that MSE enhanced adiponectin levels under both normal and obese conditions in humans [33]. Consistent with these results, we observed an increase in plasma adiponectin levels in mice fed with gnetin C- and RSV-supplemented HFCD diets compared to mice fed with a HFCD diet only (Table 2). Therefore, we assume that an increased activation of AMPK by feeding gnetin C and RSV may be partially induced by elevated adiponectin levels.

Overall, our results suggest that diet intervention with gnetin C or RSV diminished HFCD diet-induced hepatic steatosis, which imposed less injury on the liver. Consequently, fibrosis progression was prevented through the inhibition of the TGF-β1-signaling pathway. Here, we used a NAFLD model developed by feeding a HFCD diet, which is a targeted nutrient modified high fat diet. This model may not resemble human NAFLD disease pathology absolutely. Hence, further studies using other NAFLD models are required to elucidate the beneficial activity of gnetin C to a greater extent. Subsequently, human clinical trials are necessary to confirm the efficacy of gnetin C against the development of NAFLD.

## 5. Conclusions

In this study, we demonstrated for the first time that dietary supplementation with gnetin C, a RSV derivative, could successfully provide protection against NAFLD disease progression in a mice model. We also propose that gnetin C plays a greater role than RSV in reducing liver weight gain, hepatic lipid content, and fibrosis. Therefore, our findings suggest that gnetin C is a promising nutraceutical candidate for preventing NAFLD.

## Figures and Tables

**Table 1:** Effect of MSE on NAFLD phenotype.

	Control	NAFLD	0.005% MSE	0.05% MSE	0.5% MSE
BW (g)	32.8 ± 1.7	29.3 ± 3.1 *	29.4 ± 2.6	28.4 ± 2.3	25.3 ± 1.7 ^##^
Liver (g)	1.25 ± 0.08	1.46 ± 0.17 *	1.45 ± 0.11	1.46 ± 0.11	1.20 ± 0.08 ^##^
% of BW	3.83 ± 0.17	4.99 ± 0.28 **	4.95 ± 0.23	5.14 ± 0.25	4.75 ± 0.20
Plasma lipids					
TC (mg/dL)	114.6 ± 10.5	86.1 ± 7.1 **	88.0 ± 8.5	88.9 ± 6.5	79.3 ± 11.6
TG (mg/dL)	101.9 ± 20.1	89.6 ± 11.0	99.8 ± 22.9	84.8 ± 9.1	65.7 ± 11.0 ^#^
NEFA (mEq/L)	1.30 ± 0.14	1.20 ± 0.08	1.25 ± 0.11	1.11 ± 0.11	0.89 ± 0.08 ^##^
Plasma liver function markers					
AST (IU/L	25.9 ± 4.5	48.6 ± 12.2 *	43.4 ± 5.1	57.8 ± 21.2	37.5 ± 5.1
ALT (IU/L)	5.9 ± 1.4	20.9 ± 6.2 **	20.8 ± 5.1	20.3 ± 2.3	8.7 ± 2.0 ^##^
ALP (IU/L)	33.1 ± 3.7	38.3 ± 2.8 *	39.1 ± 3.4	37.3 ± 2.6	35.2 ± 3.9
Liver histology					
Steatosis	0.63 ± 0.74	2.00 ± 1.07 **	1.38 ± 0.74	0.88 ± 0.65 ^#^	0.25 ± 0.45 ^##^
Fibrosis	0.38 ± 0.51	1.88 ± 0.65 **	1.63 ± 0.51	1.25 ± 0.88	1.00 ± 0.54

BW, body weight; TC, total cholesterol; TG, triglyceride; NEFA, non-esterified free fatty acid; AST, aspartate aminotransferase; ALT, alanine aminotransferase; ALP, alkaline phosphatase. Data are represented as the mean ± SD, n = 8 in each diet; Student’s *t*-test was performed to compare between control and NAFLD for all data shown in this table. For the comparison among NAFLD, gnetin C, and RSV groups, one-way ANOVA followed by Dunnett’s test was employed for all data shown here; * *p* < 0.05, ** *p* < 0.01 vs. control; ^#^ *p* < 0.05, ^##^ *p* < 0.01 vs. NAFLD.

**Table 2:** Plasma biochemical parameters and liver lipid content.

	Control	NAFLD	Gnetin C	Resveratrol
Plasma Liver injury markers				
AST (IU/L)	57.43 ± 15.56	90.00 ± 10.61 **	83.63 ± 13.77	88.50 ± 16.58
ALT (IU/L)	2.93 ± 0.049	9.61 ± 8.03 *	3.98 ± 1.22	6.86 ± 6.72
LDH (IU/L)	146.29 ± 29.97	289.13 ± 74.65 **	220.13 ± 70.00	255.50 ± 36.08
Plasma adiponectin (ng/mL)	7187± 615	5065 ± 405 **	5943 ± 716 ^#^	5704 ± 644
Plasma lipids				
TG (mg/dL)	26.71 ± 7.71	20.13 ± 9.43	10.50 ± 1.70 ^#^	12.75 ± 3.85 ^#^
TC (mg/dL)	85.00 ± 11.90	70.13 ± 10.92 *	47.50 ± 7.62 ^##^	62.00 ± 10.21
F-CHO (mg/dL)	24.00 ± 3.16	20.63 ± 2.97	14.63 ± 2.07 ^##^	17.25 ± 2.61 ^#^
E-CHO (mg/dL)	61.00 ± 8.83	49.50 ± 8.03 *	32.88 ± 5.74 ^##^	44.75 ± 7.74
LDL-C (mg/dL)	5.00 ± 1.00	3.38 ± 0.74 *	2.13 ± 0.99 ^#^	2.63 ± 0.74 ^#^
HDL-C (mg/dL)	43.43 ± 6.85	38.00 ± 5.07	25.50 ± 4.07 ^##^	33.50 ± 5.10
NEFA (mEq/L)	0.4 ± 0.09	0.28 ± 0.04 *	0.28 ± 0.09	0.37 ± 0.04 ^#^
Liver lipids				
Total lipid (mg/g tissue)	72.28 ± 20.56	176.75 ± 36.26 **	130.67 ± 28.27	162.84 ± 52.21
TG (mg/g tissue)	21.65 ± 8.19	58.70 ± 13.54 **	49.59 ± 13.43	76.16 ± 7.34 ^#^
TC (mg/g tissue)	2.29 ± 0.91	3.29 ± 0.58 *	2.72 ± 0.50	3.67 ± 0.65
NEFA (mEq/L·g tissue)	0.047 ± 0.01	0.080 ± 0.01 **	0.055 ± 0.01 ^##^	0.062 ± 0.01 ^#^

AST, aspartate aminotransferase; ALT, alanine aminotransferase; LDH, lactate dehydrogenase; TG, triglyceride; TC, total cholesterol; F-CHO, free cholesterol; E-CHO, esterified cholesterol; LDL-C, low-density lipoprotein cholesterol; HDL-C, high-density lipoprotein cholesterol; NEFA, non-esterified free fatty acid. Data are shown as the means ± SD, n = 7–8. Student’s *t*-test was performed to compare between control and NAFLD groups for all data shown in this table except ALT, LDH, and LDL-C and these data were analyzed by the Mann–Whitney rank sum test instead of the Student’s *t*-test. For the comparison among NAFLD, gnetin C, and RSV groups, one-way ANOVA followed by Dunnett’s test was employed for all data shown here except for plasma TG and LDL-C. In the case of plasma TG and LDL-C, Kruskal–Wallis ANOVA on ranks and Dunnett’s test were applied; * *p* < 0.05, ** *p* < 0.01 vs. control; ^#^ *p* < 0.05, ^##^ *p* < 0.01 vs. NAFLD.

## Data Availability

Data is contained within the article.

## Supplementary Materials

The following supporting information can be downloaded at: https://www.mdpi.com/article/10.3390/nu15183888/s1, Table S1: Composition of experimental diets used in the current study; Table S2: Nucleotide sequences for gene specific primers; Figure S1: Weekly average food intake of mice fed control, HFCD diet, HFCD + 0.005% MSE, HFCD + 0.05% MSE, and HFCD + 0.5% MSE; Figure S2: mRNA expression of genes involved in hepatic inflammation.
