# Supplementary material for: Protective Effects of Gnetin C from Melinjo Seed Extract against High-Fat Diet-Induced Hepatic Steatosis and Liver Fibrosis in NAFLD Mice Model
Source: Nutrients. 2023 Sep 6;15(18):3888. doi: 10.3390/nu15183888 (PMC10538079; doi:10.3390/nu15183888)
Supplement: Supplementary file 1 [file nutrients-15-03888-s001.zip › nutrients-2574610-supplementary.pdf]

Supplementary Table S1. Composition of experimental diets used in the current study.

	Control	HFCD
Research Diets Inc ID	A06071314M	A06071318M
Composition (g)		
L-Cystein	4.2	4.2
L-Isoleucine	7.6	7.6
L-Leucine	15.8	15.8
L-Lysine	13.2	13.2
**L-Methionine**	**5.1**	**1.7**
L-Phenylalanine	8.4	8.4
L-Threonine	7.2	7.2
L-Tryptophan	2.1	2.1
L-Valine	9.3	9.3
L-Histidine	4.6	4.6
L-Alanine	5.1	5.1
L-Arginine	6	6
L-Aspartic Acid	12.1	12.1
L-Glutamic Acid	38.2	38.2
Glycine	3	3
L-Proline	17.8	17.8
L-Serine	10	10
L-Tyrosine	9.2	9.2
Corn Starch	502	76.2
Maltodextrin 10	130.1	100
Sucrose	68.8	172.8
Cellulose, BW200	50	50
Soybean Oil	25	25
Lard	20	177.5
Mineral Mix S10026	10	10
Dicalcium Phosphate	13	13
Calcium Carbonate	5.5	5.5
Potassium Citrate,1 H2O	16.5	16.5
Sodium bicarbonate	7.5	7.5
Vitamin Mix V10001	10	10
**Choline Bitartrate**	**2**	**0**
FD&C Yellow Dye #5	0.04	0.01
FD&C Blue Dye #1	0.01	0.04
Total	1039.35	839.55
% (w/w)		
Protein	17	21
Carbohydrate	68	43
Fat	4	24
Kcal%		
Protein	18	18
Carbohydrate	72	36
Fat	10	46
Calories per 100 g (kcal)	380	470

Supplementary Table S2. Nucleotide sequences for gene specific primers.

Gene name	Forward primer (5′-3′)	Reverse primer (5′-3′)
Eef1a1	GATGGCCCCAAATTCTTGAAG	GGACCATGTCAACAATGGCAG
Col1a1	GTACATCAGCCCGAACCCCA	GGTGGACATTAGGCGCAGGA
Smad7	GTGTTGCTGTGAATCTTACGGG	CATTGGGTATCTGGAGTAAGGAGG
TgfβI	AAGGAGACGGAATACAGGGCTT	CTGTCACAAGAGCAGTGAGCG
TgfβRI	TGGAGAAGTTTGGCGAGGCA	GCGTCCATGTCCCATTGTCTTT
TgfβRII	GTGTGACTTCGGGCTGTCCT	TCCGGGGCCATGTATCTTGC
TgfβRIII	AGTGCAAGGGGGCGTGAATA	CCGAGTAGCCATTGGTCTGGA
Mmp2	GATAACCTGGATGCCGTCGTG	GGTGTGCAGCGATGAAGATGATA
Timp2	GACTTCATTGTGCCCTGGGA	ATGGGACAGCGAGTGATCTTG
αSMA	CAGATGTGGATCAGCAAACAGGA	GACTTAGAAGCATTTGCGGTGGA
Acadl	TCCGGGAGAGTGTAAGGAAGTT	CACTTCTCCAGCTTTCTCCCAT
Acox1	TCACGCACATCTTGGATGGTAG	GTCCTCATGTTGGAAGTCTGGA
Cpt1α	CTGGATGTTTGCAGAGCACG	GACCCGAGAAGACCTTGACC
SREBP1c	AGATCTGGAAATTGCAGAGGCT	CTAAGGTGCCTACAGAGCAAGA
Chrebp	GGACAAGATCCGGCTGAACA	AACCACACACTGGGCTCTTC
Acc1	ACGTGCAATCCGATTTGTTGTC	CTGGAACATAGTGGTCTGCCA
Fasn	TTGGGTGCTGACTACAACCTCTC	CGTACACTCACTCGTGGCTCA
Dgat1	CTACCGGGATGTCAACCTGTG	CTGTAGAGACAGCTTTGGCCT
Dgat2	GCTGGCATTTGACTGGAACAC	GATGGGAAAGTAGTCTCGGAAGTAG
Mtp	TGGACGTTGTGTTACTGTGGA	CCTCTCTGTTGACCCGCATT
Hmgcr	CATGGTTCACAACAGATCAAAGATAAAT	TGCCTTCTTGGTGCACGTT
Ldlr	GGATGGCTATACCTACCCCTCAA	CCACATCGTCCTCCAGGC
Pparα	GGGAACTTAGAGGAGAGCCAAG	CAGAGCGCTAAGCTGTGATGA
Fgf21	GGTTTCTTTGCCAACAGCCA	TCCAGCAGCAGTTCTCTGAAG
Pparγ	TTGCTGTGGGGATGTCTCAC	AGATCTCCGCCAACAGCTTC
Pgc1α	CCGGAGTATGACACCGTATTTG	TCTGCTCTCACGTCTGAAGTTCTT
Sirt1	GATGACAGAACGTCACACGC	ATTGTTCGAGGATCGGTGCC
IL-6	AGAGGAGACTTCACAGAGGATGC	AATCAGAATTGCCATTGCACAAC
IL-1β	CTGTGTCTTTCCCGTGGACC	CAGCTCATATGGGTCCGACA
Cd11b	GATGGTCAGACCAACACCGAA	TTCCATTTTTGTATAAGACATCACAGC

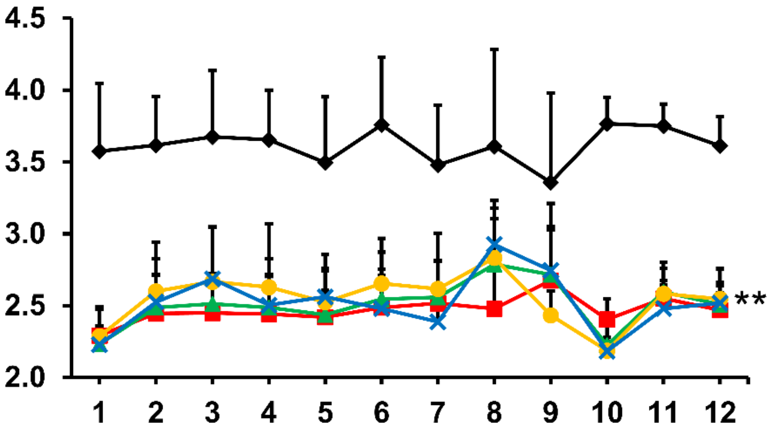

Supplementary Figure S1. Weekly average food intake of mice fed control, HFCD diet, HFCD + 0.005% MSE, HFCD + 0.05% MSE, and HFCD + 0.5% MSE. Data are shown here as means ± SD; n = 8, Student's t-Test was used to compare the control and NAFLD groups and One-way ANOVA followed by Dunnett's post-hoc analysis was carried out to make comparison among the three HFCD-fed mice groups. ** p <0.01 (vs. Control).

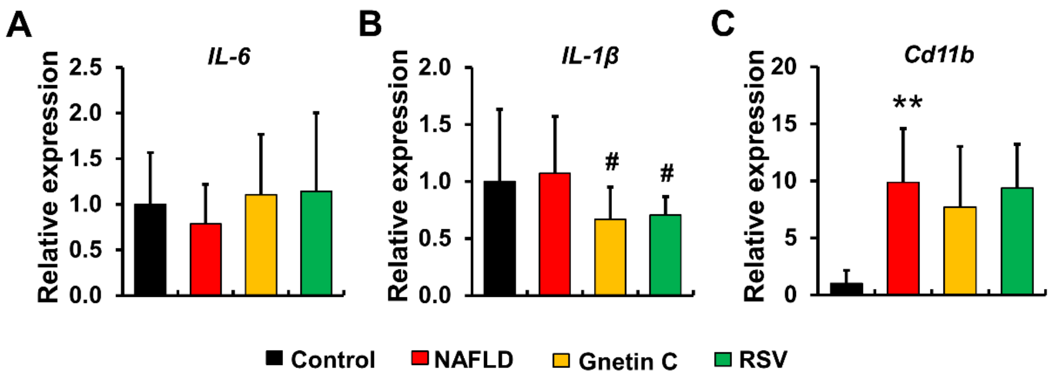

Supplementary Figure S2. mRNA expression of genes involved in hepatic inflammation: (A) *IL-6*, (B) *IL-1β* and (C) *Cd11b*. Data are shown here as means ± SD; n = 8, Student's t-Test was performed to compare control and NAFLD in case of *Cd11b*, while Mann-Whitney Rank Sum Test was used for *IL-6* and *IL-1β*. For the comparison among NAFLD, gnetin C and RSV groups, one-way ANOVA followed by Dunnett's test was employed for *Cd11b* and Kruskal-Wallis ANOVA on ranks and Dunnett's test for *IL-6* and *IL-1β;* **P < 0.01 vs. Control ; #P < 0.05 vs. NAFLD.
